# The Immediate Effect of Percussive Massage Therapy on Iliotibial Band Tightness in Field Athletes

**DOI:** 10.7759/cureus.77348

**Published:** 2025-01-12

**Authors:** Pallavi R Bhakaney, Sandhya N Towar, Tushar J Palekar, Ishan Shevate, Mrudula Sangaonkar

**Affiliations:** 1 Physiotherapy, Dr. D. Y. Patil Medical College, Hospital and Research Centre, Dr. D. Y. Patil Vidyapeeth (Deemed to be University), Pune, IND; 2 Orthopaedics, Dr. D. Y. Patil Medical College, Hospital and Research Centre, Dr. D. Y. Patil Vidyapeeth (Deemed to be University), Pune, IND

**Keywords:** athletes, exercise, iliotibial band tightness, percussive massage therapy, rehabilitation, sports

## Abstract

Background and objective

Iliotibial band tightness (ITBT) is a common knee injury that usually presents with pain and tenderness on palpation of the lateral aspect of the knee, superior to the joint line and inferior to the lateral femoral epicondyle. Athletes with ITBT typically complain of a sharp or burning pain roughly 2 cm superior to the lateral joint line. The pain may radiate proximally or distally, and in less severe cases, the pain may quickly subside upon cessation of activities. New approaches such as percussive massage therapy are used to relax the muscle tissue, reduce discomfort, and improve blood flow. It involves the use of handheld percussion devices to apply rapid vibrations and percussions to the skin and underlying soft tissue. This study aimed to examine the immediate effectiveness of percussive massage therapy on the responsiveness of pain and range of motion (ROM) in field athletes.

Methodology

Fifty athletes between the ages of 20 and 35 years were recruited for the study based on inclusion and exclusion criteria. The immediate effectiveness of percussive massage therapy (Caresmith Massage Gun, Caresmith, Mumbai, India) was assessed based on the pre-treatment and post-treatment pain levels measured on the Numerical Pain Rating Scale (NPRS) score and ROM of hip abduction and knee flexion.

Results

The results indicated a significant decrease in post-treatment pain, with the mean NPRS score decreasing from 5.18 to 2.60 immediately following the session. While the majority of participants experienced a decrease in NPRS scores, a few reported no change. The t-test for the right hip abduction ROM demonstrated a significant group effect (p<0.001). The t-test for the right knee flexion ROM also demonstrated a significant group effect (p<0.001).

Conclusions

The current study indicates that the majority of the field athletes showed a positive response to percussion massage therapy in terms of pain and ROM.

## Introduction

Iliotibial band tightness (ITBT) is a common knee injury that typically causes tenderness and pain when the lateral aspect of the knee is palpated. It is thought to be a non-traumatic overuse injury that happens frequently in runners and occurs in conjunction with underlying hip abductor weakness [1]. The iliotibial band tract is a fibrous sheath that runs the length of the lateral thigh [2]. Individuals who participate in activities involving a lot of flexion and extension, such as long-distance runners, are more likely to develop ITBT syndrome. Athletes with ITBT typically complain of a sharp or burning pain approximately 2 cm superior to the lateral joint line. In less severe situations, the pain may soon disappear if activities are discontinued. The discomfort may radiate either proximally or distally. Pain frequently arises as activities continue. Athletes often suffer from a popping sound on the lateral aspect of their knees when engaging in certain sports.

Conventional physiotherapy treatment involves a myofascial release technique, using a foam roller to release the tightness of the muscles, and exercises to strengthen the abductor muscles [3]. However, relatively new approaches such as percussive massage therapy are also used to relax the muscle tissue, reduce discomfort, and improve blood flow. It involves the use of handheld percussion devices to apply rapid vibrations and percussions to the skin and underlying soft tissue [4]. This therapy is based on tapotement massage techniques and mechanical vibration, and it has been found to promote vasodilation, stimulate cutaneous reflexes, and elicit neurological and physiological responses [5]. It can be used both as a warm-up and recovery tool to enhance muscle flexibility and reduce soreness.

Mechanoreceptors, such as Pacinian corpuscles and Ruffini receptors, are essential in ensuring the therapeutic benefits of percussion therapy [6]. High-frequency vibrations or low-amplitude frequencies can activate these receptors, influencing sympathetic activity and leading to positive results. However, it is important to note that different receptors may react diversely to mechanical percussion, highlighting the need for tailored treatments based on individual characteristics and body regions. While this method shows promising results, understanding the specific impact on mechanoreceptors and refining treatment parameters are key for successful implementation in rehabilitation and recovery. Recent studies have suggested a growing trend in the use of handheld percussion devices by physical therapists and athletes to address musculoskeletal issues [7]. This study aims to examine the immediate effectiveness of percussive massage therapy on the responsiveness of pain and range of motion (ROM) in field athletes.

## Materials and methods

The study was conducted as an interventional study, spanning the period from January 2024 to July 2024 at Dr. D. Y. Patil College of Physiotherapy, and involved convenient sampling to select a sample size of 50 athletes, determined by G*power version V3.1.9.4 software, aiming for a 95% confidence level and 80% power, based on an effect size of 0.5. Informed written consent was obtained from all participants before their involvement in the study. The sample population consisted of athletes aged 20-35 years, both male and female, experiencing slight pain on the iliotibial band after 1-2 hours of continuous practice, rated 6/10 on the Numerical Pain Rating Scale (NPRS) scale, willing to participate, and with positive Ober’s test. Exclusion criteria included those with pain exceeding 7 on the NPRS scale due to contraindications, previous musculoskeletal injuries of the hip or knee, neurological diseases, recent trauma, skin rashes, open wounds, and inflammatory arthritis.

**Figure 1:**
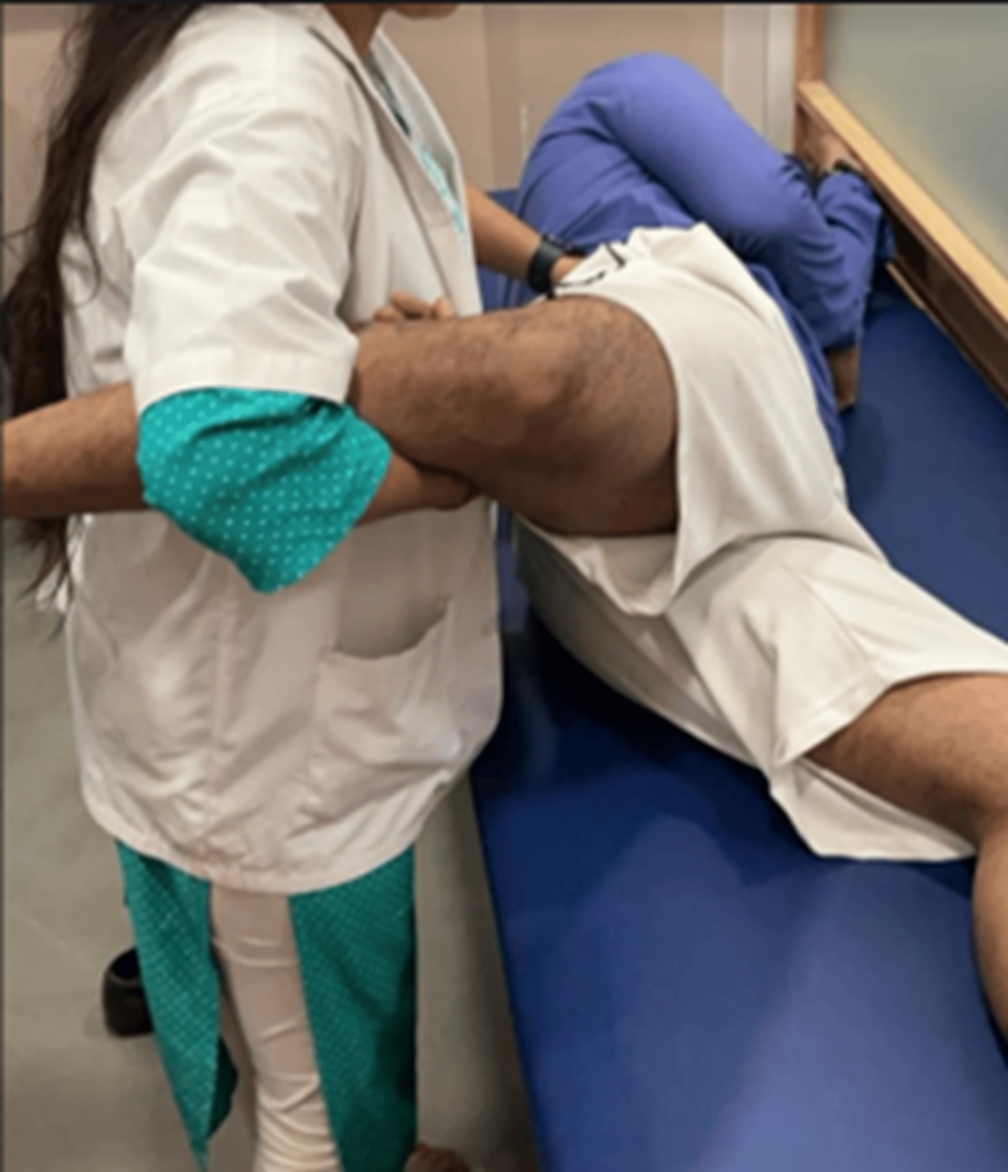
The Ober test performed by the therapist with the athlete in the side-lying position with pelvis stabilized and knee properly supported

Pre and post-treatment measurements of pain and ROM were recorded for each participant, before and immediately after the session. Screening of the athletes was done using the Ober test. Both the limbs of the athletes were thoroughly assessed for ITBT using the test [8]. To be tested for ITBT, the athlete was positioned in the side-lying position, with the affected side upwards. The knee of the athlete on the downward side was flexed to flatten the lumbar spine. The gluteal muscles of the athlete were also stabilized to avoid any trick movement. The hip joint was extended and abducted, and slowly the hip was adducted. The test was considered positive when the leg remained in the abducted position, as it implies tightness in ITB (Figure 1).

**Figure 2:**
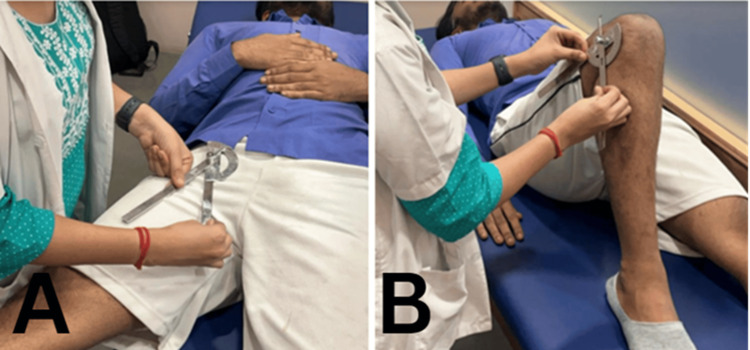
Assessment of ROM using universal goniometer (A) Hip abduction ROM in the supine lying position. (B) Knee flexion ROM in the supine lying position ROM: range of motion

All the athletes recruited for the study with a positive Ober test were checked for pain perceived by the athlete on NPRS as well as ROM of hip abduction and knee flexion (Figure 2).

**Figure 3:**
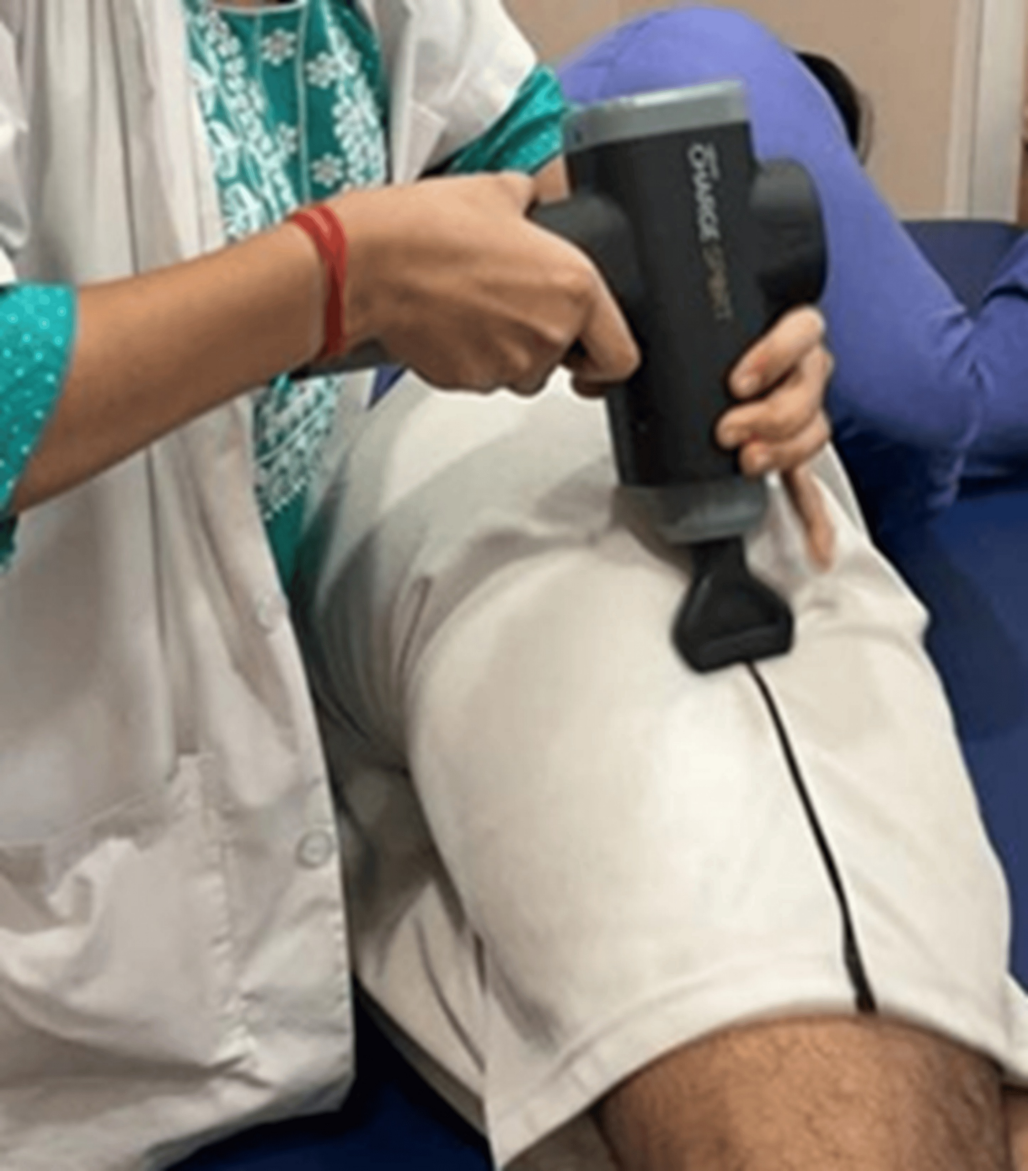
Application of the Theragun in a side-lying position, with the affected side up along the length of ITB ITB: iliotibial band

Application using Theragun was done, bilaterally, with the patient in a side-lying position and the affected side up, hip in a neutral position, and knee in an extension position (Figure 3). The Theragun was applied along the length of ITB with a frequency of 2200 repetitions per minute (RPM), with a round head for about 15 minutes. Immediately after the session, pain was assessed on NPRS, and hip abduction and knee flexion ROM were assessed. Data collection was conducted meticulously, ensuring accuracy and reliability. Subsequently, the collected data were analyzed, and results were calculated. Means were calculated using SPSS Statistics version 25 (IBM Corp., Armonk, NY). A paired t-test was used to analyze pre and post-treatment dependent variables. Later, using the previously derived means, pre and post-groups were compared using the paired t-test. Ethical standards and guidelines were strictly adhered to throughout the research process.

## Results

Sports-wise distribution of the athletes was as follows: 19 cricketers (38%), 15 footballers (30%), 13 badminton players (26%), two basketball players (4%), and one karate player (2%). The t-test for pain levels demonstrated a significant group effect (p<0.001). Post-treatment analyses revealed that individuals in the pre-treatment pain group reported significantly higher mean pain scores (5.18) compared to those in the post-treatment group (2.60) (Table 1).

**Table 1:** The pre- and post-treatment NPRS scores NPRS: Numerical Pain Rating Scale

		Mean	Standard deviation	Standard error	P-value
Pain	Pre-treatment	5.18	0.94	0.13	<0.001
	Post-treatment	2.60	0.88	0.12	

The t-test for the right hip abduction ROM demonstrated a significant group effect (p<0.001). Post-treatment analyses revealed that individuals in the pre-treatment group reported significantly decreased mean degree ROM scores [right (40.80) and left (40.48)] compared to those in the post-treatment group [right (45.64) and left (45.38)] (Table 2)

**Table 2:** The pre- and post-treatment hip abduction ROM scores ROM: range of motion

		Mean	Standard deviation	Standard error	P-value
Hip abduction (right)	Pre-treatment	40.80	6.20	0.88	<0.001
	Post-treatment	45.64	6.48	0.92	

The t-test for the right knee flexion ROM demonstrated a significant group effect (p<0.001). Post-treatment analyses revealed that individuals in the pre-treatment group reported a significant decrease in mean degree ROM scores [right (122.66) and left (124.36)] compared to those in the post-treatment group [right (128.12) and left (129.68)] (Table 3).

**Table 3:** The pre- and post-treatment knee flexion ROM scores ROM: range of motion

		Mean	Standard deviation	Standard error	P-value
Knee flexion (right)	Pre-treatment	122.66	13.27	1.88	<0.001
	Post-treatment	128.12	13.94	1.97	

## Discussion

The study aimed to document the effectiveness of a single session of percussive massage therapy in ITBT in the athletic population. The results indicated a significant decrease in post-treatment pain, with a mean NPRS score reduction from 5.18 to 2.60 immediately following the session. While the majority of participants experienced a decrease in NPRS scores, a few reported no change. These varying responses align with the pain gate theory, which suggests that sensory stimulation can either activate non-nociceptive fibers, resulting in pain reduction, or nociceptive fibers, exacerbating pain and soreness. The mechanism behind this effect could involve the activation of mechanoreceptor nerve endings, leading to increased soreness immediately after therapy. Additionally, the high frequency of the percussion therapy device might contribute to these outcomes.

A study by Sams et al. (2023) on the effect of percussive therapy on musculoskeletal performance and experiences of pain in a literature review concluded that patients have reported a reduced perception of pain [9]. Furthermore, the present study observed an increase in ROM following treatment, with notable improvements in hip abduction and knee flexion, particularly in the latter. Although the reasons behind these changes were not fully elucidated, previous research suggests that percussion therapy may lead to biomechanical alterations, such as reduced muscle compliance, increased blood flow, reduced pain perception, and psychological relaxation, as documented by Alvarado et al. (2022) [10]. Additionally, the application of pressure and friction during percussive massage could affect tissue viscosity, thereby reducing resistance to movement. This could explain the observed increase in the ROM in the population.

Moreover, research indicates that percussion therapy may influence motor unit functioning and joint discomfort through changes in fascial properties and sensory input. By targeting the fascia and muscle spindles, percussion treatment may alleviate stiffness and restore proprioception. Different mechanical forces, including compression, tension, torsion, and shear, can mechanically soften tissues, making them more pliable. Additionally, studies have demonstrated an increase in intramuscular volume with passive stretch tension, which could contribute to ROM improvements [11]. Research on posterior shoulder muscles indicated an increase in the internal rotation ROM following percussion therapy [12]. Hence, percussion therapy shows promising effects in reducing pain and improving ROM, possibly through various biomechanical, physiological, and neurological mechanisms [13,14].

Limitations

This study has a few limitations, primarily its small sample size, which affects the generalizability of our findings. Studies with larger sample sizes are required to gain a better understanding of the topic. Also, the study duration was relatively small, which restricted the scope of research. Further research is needed to better understand the effects of this technique and optimize its application in rehabilitation settings.

## Conclusions

The use of handheld percussion device therapy is an emerging practice among both athletes and therapists. The current study indicates that the majority of the athletes had a positive response to percussion therapy in terms of pain and ROM. The subjects with ITBT benefitted from percussion therapy as it showed a significantly higher decrease in pain as noted by the NPRS score and the increase in ROM.

## Figures and Tables

**Figure 4:**
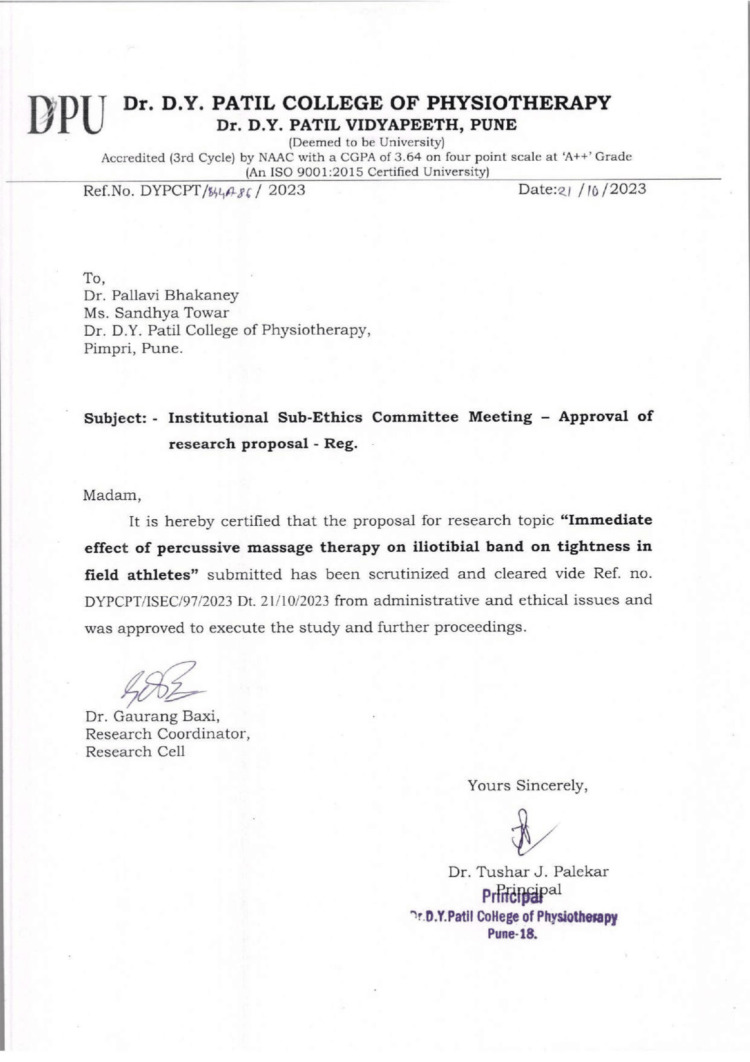
Institutional Ethical Committee approval letter
